# Directed transport of fractional‑order asymmetrically coupled particle chains in ratchet potential

**DOI:** 10.1371/journal.pone.0349159

**Published:** 2026-10-29

**Authors:** Zhe Tu, Tao Yu

**Affiliations:** 1 Business School, Zhejiang Wanli University, Ningbo, Zhejiang, China; 2 College of Mathematics, Sichuan University, Chengdu, Sichuan, China; Donghua University, CHINA

## Abstract

This work constructs a fractional-order model to describe the dynamics of particle chains with asymmetric coupling subject to non-uniform viscoelastic media and an asymmetric periodic potential. The effects of multiple control parameters on the collective directed motion are systematically investigated. Numerical results reveal that the fractional-order coupled system can achieve directed transport in the absence of external forces and noise driving. Under suitable coupling strengths, the directed transport velocity of the particle chain generally rises as the fractional order increases. We also find that the average velocity of the particle chain exhibits generalized resonance responses with varying system parameters, featuring single-peak, double-peak and multi-peak resonant behaviors. Such resonant behaviors are sensitive to system parameters and originate from the competition between fractional power-law memory and interparticle coupling. Reverse directed transport can be triggered under appropriate parameter combinations, arising from the interplay of asymmetric coupling, medium memory and ratchet potential. Furthermore, under appropriate system parameters, noise can enhance the directed transport velocity, manifesting stochastic resonance in the system.

## Introduction

Directed transport of energy and matter in nonlinear systems exhibits extensive application potential, including transport process regulation, fine particle separation and controllable biological function realization [1–4]. Accordingly, the directed transport performance of molecular motors has attracted increasing attention from researchers across diverse disciplines [5–12]. Growing research confirms that molecular motors usually operate in collective modes. Intermotor interactions are inevitable and profoundly affect macroscopic transport behaviors [13–18]. For instance, Guo et al. [13] verified that strong inter-particle coupling can effectively promote directed transport. Zhang et al. [14] demonstrated that transport current varies non-monotonically with coupling strength. Downton et al. [15] found that internal interactions within motor chains significantly reduce the transport efficiency of multi-motor systems compared with individual motors. Zhu et al. [16] pointed out that topological lattice structures dominate particle motion directions, while particle number density also plays a crucial role in modulating directed transport. Roostalu et al. [17] revealed that the motor protein Cin8 can reverse the transport direction through intermolecular coupling, with their findings published in *Science*. Their experiments further demonstrated that such directional switching originates from asymmetric mechanical interactions between adjacent motor units, which serves as a biological prototype of the asymmetric unidirectional coupling scheme explored in this work. Such unidirectional coupling widely exists in many real-world systems, such as signal transmission in neural networks, traffic flow, electrical circuit experiments and coupled Josephson junctions [4].

Existing studies have explored a broad range of dynamic processes and engineering optimization problems related to transport and signal regulation. Representative works include integer-order numerical simulations of microbial transport, experimental optimization of nanoparticle-based thermal systems, and Gaussian regression-enabled wireless positioning technologies [19–21]. These studies provide reliable numerical frameworks and practical references for system dynamic analysis and parameter optimization. Nevertheless, most classical models are established within integer-order theoretical frameworks and fail to characterize the inherent memory properties of complex media and the collective coupling behaviors of particle clusters.

Most currently available dynamic models, including the above investigations, rely on classical integer-order formulations. Integer-order equations can accurately describe linear macroscopic dynamic responses. They cannot capture the temporal memory and long-range spatial correlation inherent in heterogeneous complex media. At present, research on directed transport in coupled systems is predominantly confined to integer-order dynamical systems. accumulating evidence indicates that particle motion in physical, biochemical and viscoelastic systems presents obvious temporal and spatial nonlocality, namely the memory effect. Fractional calculus naturally incorporates time memory and spatial correlation characteristics. It is therefore highly suitable for describing physical, chemical and biological phenomena with memory, heredity and path-dependent features [22–27]. These fractional-order studies can be broadly grouped into two categories. The first covers stochastic resonance and noise-induced dynamics: Refs [22–24] examines resonance in fractional oscillators with random mass, random damping and random intrinsic frequency. The second concerns directed transport and its theory: Refs [25] and [26] investigate directional transport of fractional coupled Brownian motors in periodic potentials, and Ref [27] establishes the large deviation principle for slow-fast systems with mixed fractional Brownian motion. Collectively, these works demonstrate the advantages of fractional frameworks in capturing memory and nonlocal effects beyond integer-order models.

In microscopic fractal structures of complex media, anomalous diffusion is commonly observed, deviating from the classical Einstein diffusion relation $\langle x^{2}\rangle =2Dt$. Obeying the power‑law scaling $\langle x^{2}\rangle =2Dt^{\alpha }$, such diffusion corresponds to fractional Brownian motion rather than conventional Markovian random processes. Since fractional Brownian motion encodes historical‑memory information, time‑fractional differential operators are employed to describe the memory‑dependent damping effect, yielding the fractional Langevin equation $\ddot{x}+\gamma \frac{d^{\alpha -1}}{dt^{\alpha -1}}\dot{x}=\Gamma (t)$.

Viscoelastic media serve as typical complex materials with mechanical properties between ideal elastic solids and Newtonian fluids. Their stress–strain evolution exhibits obvious time-memory characteristics [28]. Experimental observations show that the stress relaxation and creep behavior of most viscoelastic materials follow power-law time-dependent rules. To accurately describe such power-law mechanical responses, Bagley introduced fractional-order derivatives into the constitutive equations of viscoelastic materials, forming a classic framework for characterizing medium memory effects [28,29]. In addition, Adolfsson et al. [30] found that fractional-order models are capable of accurately describing the dynamic characteristics of numerous complex viscoelastic materials using relatively few parameters, while integer-order models need multiple derivative terms and additional material parameters.

Moreover, fractional calculus has been widely adopted in the research of coupled nonlinear systems. Relevant studies have developed fractional-order controllers together with disturbance observers to enhance system robustness against noise and parameter uncertainties, utilizing fractional operators to capture memory effects. For coupled control systems built upon fractional dynamic descriptions, fractional memory effects and generalized resonance could potentially come into play and may alter controller stability characteristics. In addition, data-driven approaches provide another paradigm for analyzing complex systems. Numerical data collected from fractional dynamic simulations can support the construction of approximate models for follow-up research [31–33].

Different from conventional integer-order models and engineering-oriented optimization studies, this paper adopts fractional calculus to describe the power-law memory characteristics of heterogeneous media. Without external driving forces, a fractional-order dynamic model of asymmetrically coupled particle chains under asymmetric ratchet potentials is constructed. The collective directed transport mechanism is systematically investigated. Numerical simulations based on the fractional difference method are performed to explore the influences of key system parameters on particle chain transport velocity. The numerical results show that the fractional-order asymmetric coupling system can sustain spontaneous directed transport even in the absence of external force and noise excitation. Transport velocity increases monotonically with the fractional order. Under a fixed fractional order, the average transport velocity presents generalized resonance responses to coupling strength and potential parameters, and reverse transport can be triggered under specific parameter combinations. Furthermore, stochastic resonance behaviors emerge in particle chain average velocity when noise is introduced into the system. In summary, the main distinct contributions of this work are as follows: (i) a fractional-order dynamic model for asymmetrically coupled particle chains is established to capture the power-law memory effect omitted in traditional integer-order ratchet systems; (ii) we reveal directed transport in the absence of external force and noise; (iii) we characterize the generalized resonance, stochastic resonance and reverse transport behaviors governed by system parameters.

## Model

In non-uniform viscoelastic media, the damping force acting on particles generally depends on their velocity history and displays memory features. Here we adopt the overdamped approximation and neglect inertial terms. For microscale particles moving in viscoelastic environments, the Reynolds number is far less than unity, so viscous dissipation dominates particle dynamics and inertial effects can be safely omitted. This simplification is widely accepted for modeling ratchet transport and molecular motor systems at microscale [34]. Neglecting inertial terms, the dynamics of particles with nearest-neighbor coupling in a periodic potential landscape are governed by the following nonlinear integro-differential equation:


$$\begin{array}{c} \int_{0}^{t}\gamma (t-\tau ){\dot{x}}_{i}(\tau )d\tau =-\frac{dV(x_{i})}{dx_{i}}+g(x_{i-1},x_{i},x_{i+1})+\sqrt{2D}\xi_{i}(t),\;i=1,2,\cdots ,N \end{array}$$ (1)

Here, $x_{i}(t)$ stands for the displacement of the *i*-th particle. The function γ(t) is the damping kernel characterizing the memory effect of heterogeneous media. *V*(*x*) represents the periodic potential function. $g(x_{i-1},x_{i},x_{i+1})$ denotes the nearest-neighbor coupling function for the i-th particle. *D* is the noise intensity. $\xi_{i}(t)$ refers to zero-mean Gaussian white noise that satisfies:


$$\begin{array}{c} \langle \xi_{i}(t)\rangle =0,\;\langle \xi_{i}(t)\xi_{j}(s)\rangle =\delta_{ij}\delta (t-s). \end{array}$$ (2)

This paper investigates a typical form of unidirectional coupling widely found in many systems, including signal transmission in neural networks and traffic flow. Specifically, the nearest-neighbor coupling function is defined as:


$$\begin{array}{c} g(x_{i-1},x_{i},x_{i+1})=k(x_{i+1}-x_{i}-a),\;i=1,2,\cdots ,N \end{array}$$ (3)

Here, *k* denotes the coupling coefficient, which characterizes the strength of unidirectional coupling between neighboring particles. The parameter *a* represents the equilibrium spring length between adjacent particles.

We employ periodic boundary conditions [35]. Specifically, we assume


$$\begin{array}{c} x_{N+1}=x_{1}+Na \end{array}$$ (4)

Accordingly, the coupling force acting on the *N*-th particle is expressed as


$$\begin{array}{c} g(x_{N-1},x_{N},x_{N+1})=k(x_{1}+Na-x_{N}-a) \end{array}$$ (5)

In this work, the periodic potential adopts the form of a sinusoidal ratchet potential, namely:


$$\begin{array}{c} V(x)=-\frac{1}{2}U_{0}\left[\sin(2\pi x/L)+\frac{\Delta }{4}\sin(4\pi x/L)\right] \end{array}$$ (6)

Here, *U*_0_ denotes the barrier height. *L* represents the spatial period of the ratchet potential and is fixed at *L* = 1. The parameter Δ is the asymmetry parameter characterizing the asymmetry degree of the ratchet potential. When Δ>0, the potential barrier inclines to the right. When Δ<0, it inclines to the left. The potential becomes fully symmetric for Δ=0. This feature has been elaborated in Ref [25].. Therefore, we mainly investigate the case of Δ≠0 for the ratchet potential. A schematic illustration of the ratchet potential is shown in Fig 1.

**Fig 1:**
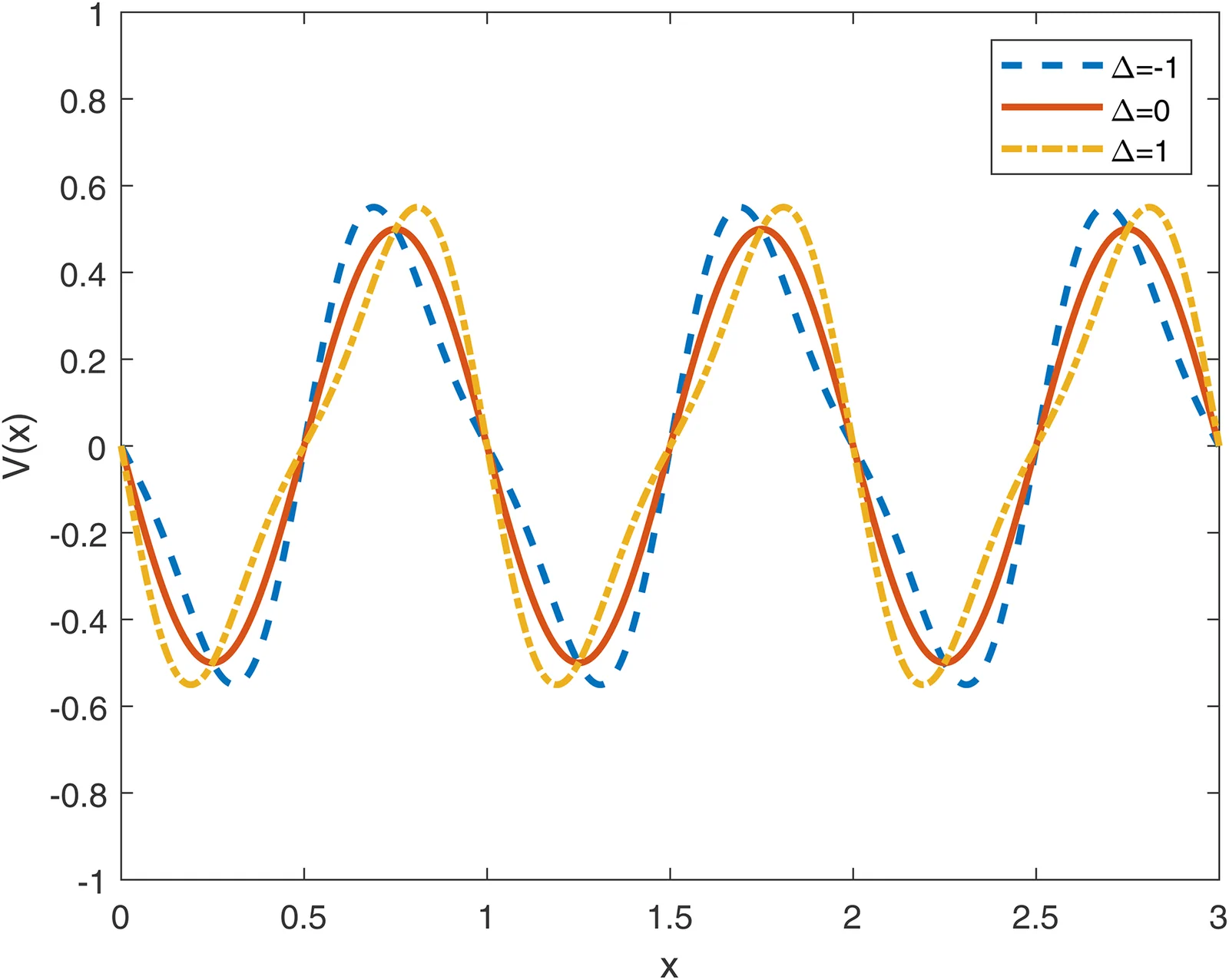
Schematic diagram of the ratchet potential.

In many physical and biochemical processes, heterogeneous media exhibit power-law memory associated with particle velocity. Memory effects become more pronounced for events occurring closer to the present, and historical velocity exerts a greater impact on particle motion [25,26,36]. Accordingly, we adopt a power-law expression for the damping kernel function:


$$\begin{array}{c} \gamma (t)=\frac{1}{\Gamma (1-\alpha )}|t{|}^{-\alpha },\;0<\alpha <1 \end{array}$$ (7)

Based on Caputo’s definition of the fractional derivative [37], we obtain


$$\begin{array}{c} \frac{d^{\alpha }x_{i}}{dt^{\alpha }}=\frac{1}{\Gamma (1-\alpha )}\int_{0}^{t}\frac{{\dot{x}}_{i}(u)}{(t-u{)}^{\alpha }}du,\;\alpha \in (0,1) \end{array}$$ (8)

This expression reveals that the viscous medium possesses power-law memory. The fractional order α characterizes the strength of such memory, as illustrated in Fig 2. Specifically, when α=1, the fractional-order system reduces to an integer-order system. Under this condition, the damping force acting on the particle depends solely on the instantaneous velocity at the current time. In other words, the memory property of damping vanishes.

**Fig 2:**
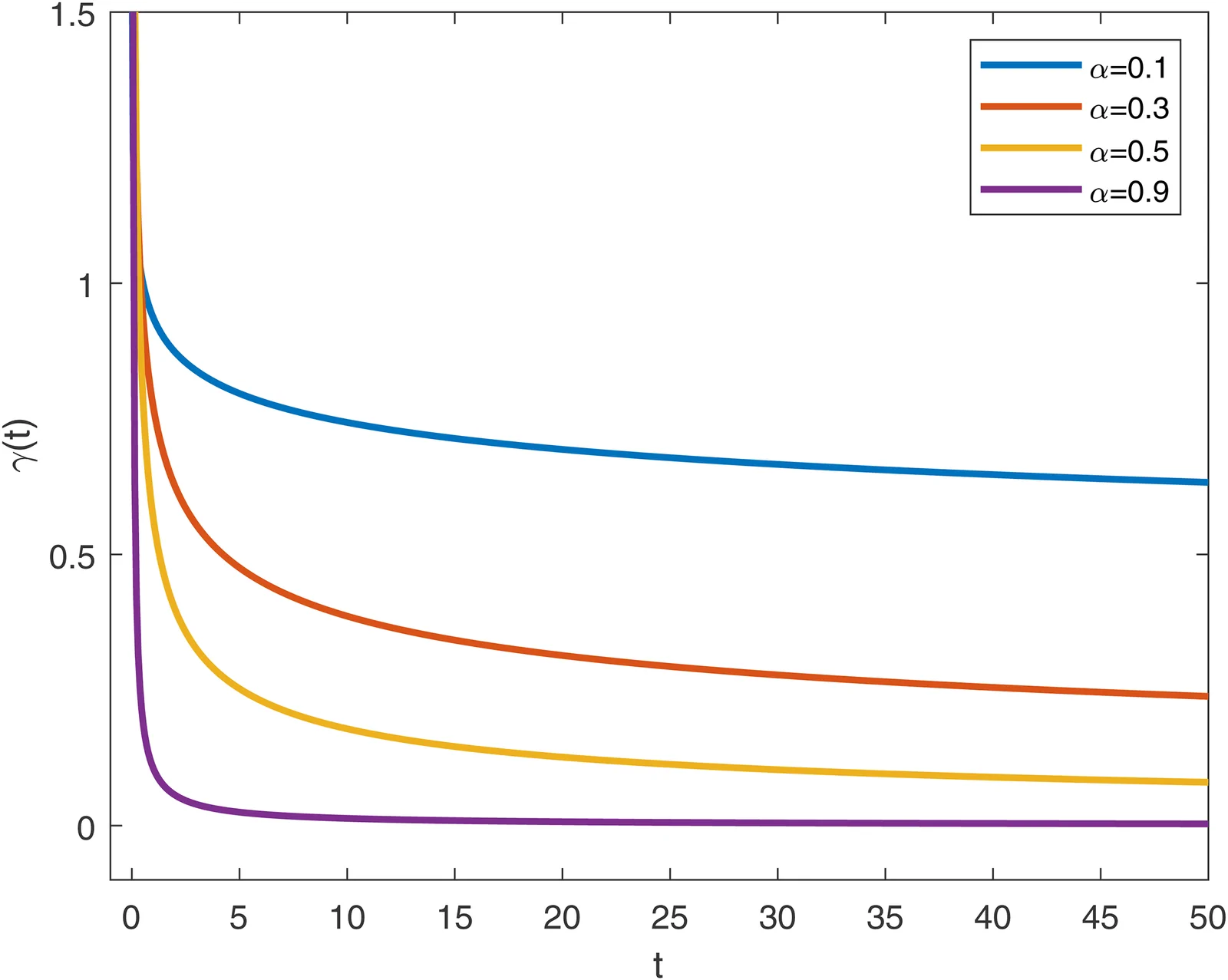
Schematic diagram of the damping kernel function.

Combined with the definition of Caputo fractional calculus and the nearest-neighbor coupling function (Eq (3)), Eq (1) can be rewritten as a fractional equation:


$$\begin{array}{c} \frac{d^{\alpha }x_{i}}{dt^{\alpha }}=-\frac{dV(x_{i})}{dx_{i}}+g(x_{i-1},x_{i},x_{i+1})+\sqrt{2D}\xi_{i}(t),i=1,2,\cdots ,N,0<\alpha <1 \end{array}$$ (9)

## Numerical simulation and analysis

### Numerical simulation method

The numerical simulation method adopted in this work is the Grünwald–Letnikov discretization, which is derived from the power series expansion of fractional operators [38]. Note that the Grünwald-Letnikov scheme is originally constructed for the Riemann‑Liouville fractional derivative. It can be used to accurately approximate the Caputo fractional operator under zero initial conditions, where the Caputo and Riemann‑Liouville definitions are mathematically equivalent. It is worth noting that radial basis function (RBF) meshless methods are powerful for multi-dimensional fractional partial differential equations on complex domains [39,40]. Nevertheless, the Grünwald–Letnikov scheme is convenient and efficient for the one-dimensional particle-chain fractional dynamics in this work.

Let $x_{i}(t_{n})$ denote the displacement of the *i*-th particle at time $t_{n}=n\Delta t$. For sufficiently small time step Δt, the numerical discretization of system (Eq (9)) reads:


xi(tn)=−∑m=1n(−1)m(αm)xi(tn−m)+fi(tn−1)Δtα,i=1,2,⋯,N,0<α<1 (10)

where


$$\begin{array}{c} f_{i}(t_{n-1})=-\frac{dV(x_{i}(t_{n-1}))}{dx_{i}}+g(x_{i-1}(t_{n-1}),x_{i}(t_{n-1}),x_{i+1}(t_{n-1}))+\sqrt{2D}\xi_{i}(t_{n-1}). \end{array}$$ (11)

The stochastic term $\xi_{i}(t_{n-1})$ is generated following the procedure described in [41].

This explicit fractional Euler scheme originates from the Grünwald-Letnikov fractional difference and possesses a local truncation error 𝒪(Δt), namely first-order temporal accuracy [36,37]. Mathematically, when α=1 the discrete scheme of Eq (10) formally reduces to the standard Euler difference scheme for integer-order differential equations. The truncation error estimate and the consistency property recovered in the integer-order limit demonstrate the mathematical consistency of this discretization. The Grünwald–Letnikov-based explicit fractional Euler scheme has been widely validated and successfully applied to simulate fractional stochastic dynamical systems with memory effects in previous studies [36,37].

The average directed transport velocity of the particle chain in the coupled system can be defined as [35]:


$$\begin{array}{c} v=\lim_{t\to \infty }\frac{1}{N}\sum_{i=1}^{N}v_{i}=\lim_{t\to \infty }\frac{1}{N}\sum_{i=1}^{N}\frac{x_{i}(t)-x_{i}(t_{0})}{t-t_{0}} \end{array}$$ (12)

Here, *t*_0_ and *t* denote the initial and terminal times of particle motion. The symbol $v_{i}$ represents the average velocity of the *i*-th particle. The particle chain average velocity characterizes the macroscopic overall motion trend of the particle ensemble. Therefore, this quantity is adopted as the core research variable in this work to investigate the directed transport behavior of the fractional-order coupled system under an asymmetric periodic potential, as well as to explore the parameter-dependent transport characteristics via numerical simulations.

In the numerical simulations, we adopt $v\approx \frac{1}{N}\sum_{i=1}^{N}\frac{x_{i}(T)-x_{i}(t_{0})}{T-t_{0}}$ to approximately calculate the mean transport velocity *v*, where *T* is the total simulation time. To quantitatively evaluate the synchronization degree of the particle chain, we further define the variance of particle average velocity $S_{v}$ as follows:


$$\begin{array}{c} S_{v}=\frac{1}{N}\sum_{i=1}^{N}(v_{i}-v{)}^{2} \end{array}$$ (13)

To determine reliable and convergent simulation parameters, we systematically analyze the temporal convergence characteristics of the average velocity *v* and its variance $S_{v}$ under different particle numbers, as illustrated in Fig 3 and Fig 4.

**Fig 3:**
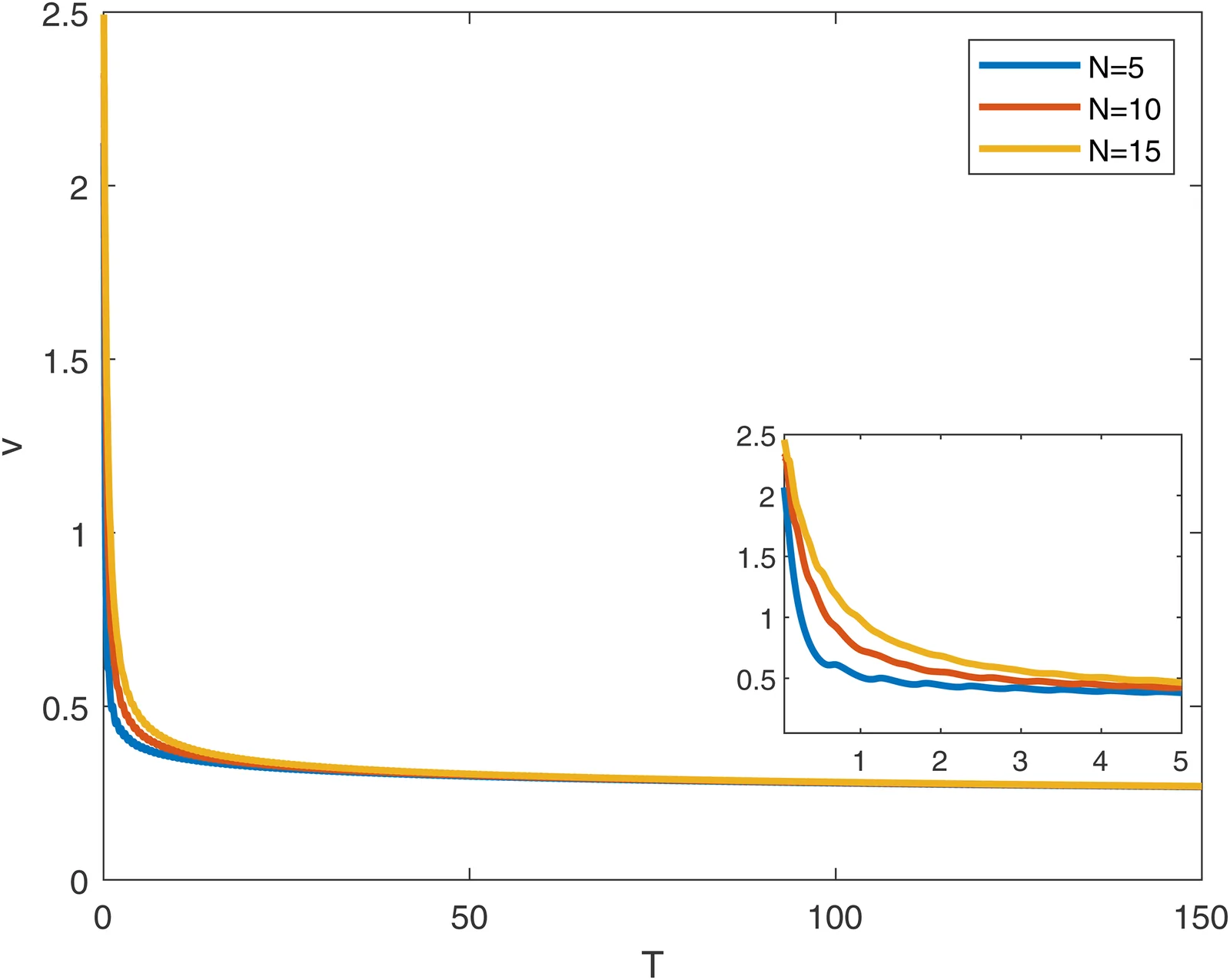
Average velocity of particle chains vs. simulation time under different particle numbers. Other parameters are set as follows: $\alpha =0.9,k=10,U_{0}=0.5,\Delta =1,a=0.2,D=0$.

**Fig 4:**
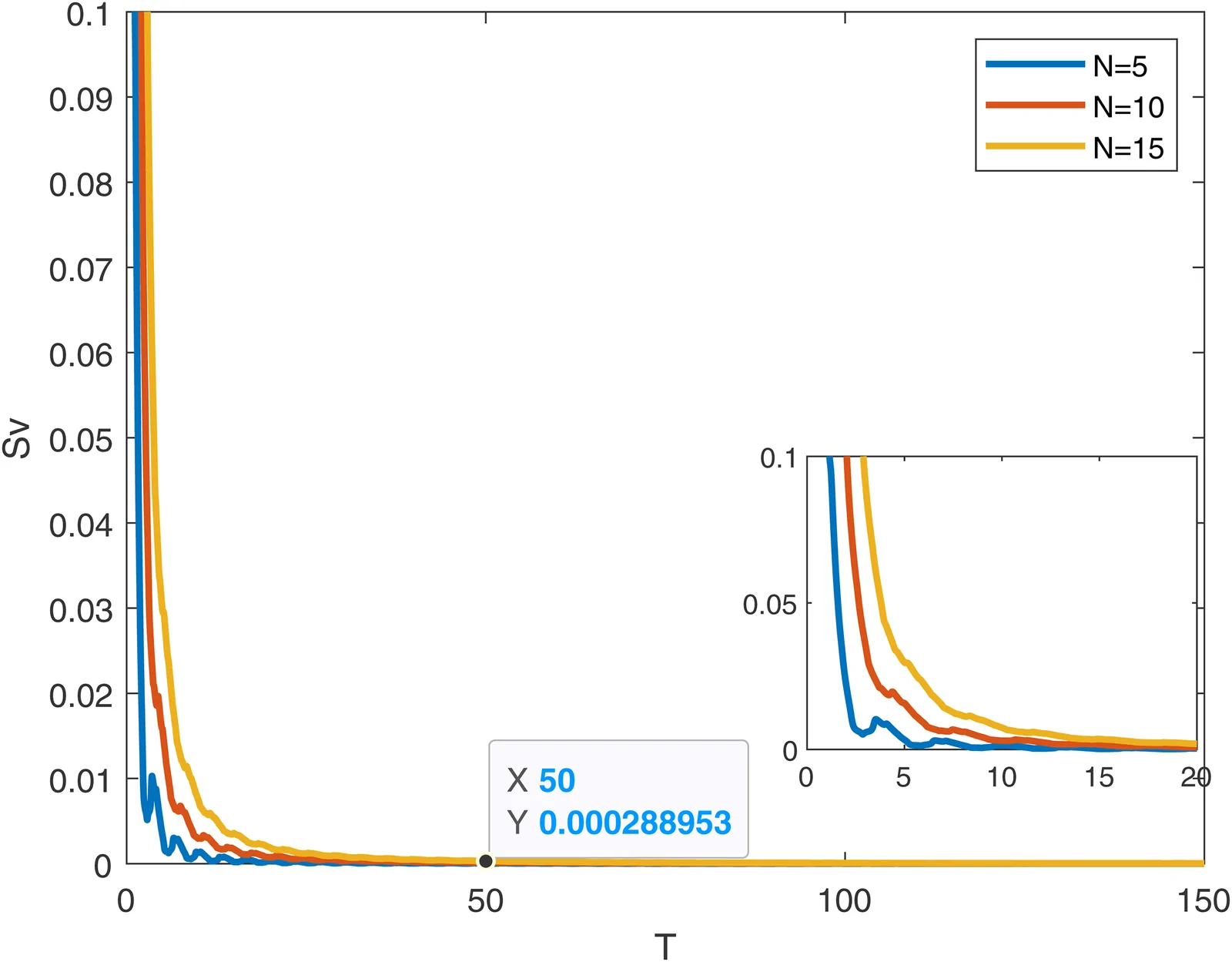
The variance of the average velocity of the particle chain vs. simulation time under different particle numbers. Other parameters are set as follows: $\alpha =0.9,k=10,U_{0}=0.5,\Delta =1,a=0.2,D=0$.

As can be seen in Fig 3, the mean transport velocity of the particle chain converges rapidly to a stable constant value, and the particle number only affects the transient convergence rate rather than the final steady-state velocity. As depicted in Fig 4, the velocity variance $S_{v}$ decreases to the order of 10^–4^ at the simulation time *T* = 50 s, indicating that the velocity differences among individual particles are sufficiently small and the particle chain achieves a stable synchronized state. In addition, the synchronization time is positively correlated with the number of particles, requiring longer evolution time for systems with more particles to reach synchronization.

Collectively, Fig 3 and Fig 4 demonstrate that the particle chain can rapidly achieve synchronized steady-state motion with convergent average velocity. Accordingly, the transient influence of initial conditions is completely decayed and thus can be neglected for steady-state analysis.

Throughout all major simulations in this work, zero initial particle positions $x_{i}(0)=0$ are uniformly adopted. To validate the robustness of our results, we perform supplementary tests with small random perturbations imposed on the zero initial positions. The results indicate that different perturbed initial positions only affect the transient synchronization time of the particle chain, while the mean average velocity and the directed transport behaviors remain unchanged. For computational efficiency and without loss of generality, we fix $x_{i}(0)=0$ for all numerical examples presented in this manuscript.

In summary, the simulation parameters in this paper are fixed as follows: the total simulation time *T* = 50 s, the sampling time step Δt=0.01, the initial particle position $x_{i}(0)=0$. Unless otherwise specified, the system parameters are set as follows: the fractional system order α=0.9, the particle number *N* = 10, the coupling coefficient *k* = 10, the potential barrier height *U*_0_ = 0.5, the potential asymmetry parameter Δ=1, and the equilibrium spring length *a* = 0.2.

Before presenting the numerical results, we clarify the resonance-related terminology adopted in this work. Stochastic resonance refers to the phenomenon that the average transport velocity exhibits non-monotonic variation with noise intensity. For noise-free systems, if the average transport velocity shows non-monotonic dependence on deterministic parameters (e.g., coupling strength, asymmetry parameter), we term this behavior generalized resonance.

### Deterministic directed transport in the absence of noise (D = 0)

#### Particle chain displacement.

Fig 5 displays the time evolution of displacement $x_{i}(t)$ for each particle within the chain under three sets of control parameters.

**Fig 5:**
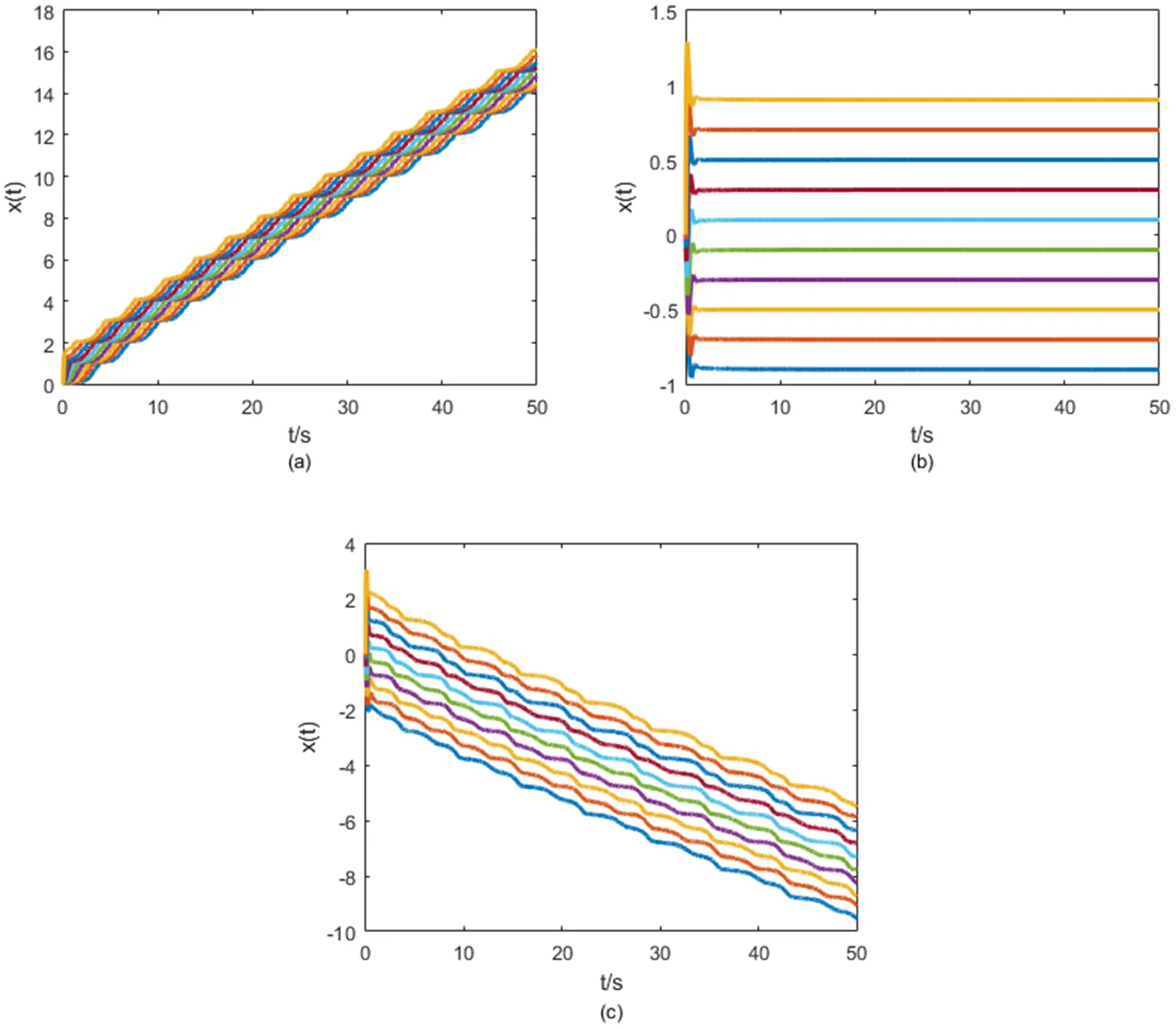
Displacement plot *x*(*t*) of particle chains under different parameters. (a): $\alpha =0.9,k=10,U_{0}=0.5,\Delta =1,a=0.2,D=0$ and (b): $\alpha =0.9,k=10,U_{0}=0,\Delta =1,a=0.2,D=0$ and (c): $\alpha =0.9,k=20,U_{0}=0.5,\Delta =1,a=0.45,D=0$.

Subplot (a) corresponds to the configuration $\alpha =0.9,k=10,U_{0}=0.5,\Delta =1,a=0.2,D=0$. It can be observed that all particles move persistently toward the positive direction. The displacement curves increase linearly over time with small periodic oscillations, indicating stable positive directed transport. The particles maintain nearly uniform separation throughout the simulation, which demonstrates synchronized collective motion. Notably, this deterministic directed transport emerges without external forcing or noise, originating from the combined effect of interparticle asymmetric coupling and the asymmetric ratchet potential.

Subplot (b) illustrates the case where the periodic potential vanishes (*U*_0_ = 0), while other parameters remain unchanged. The particle displacement quickly saturates after a short transient stage, and particles merely oscillate around fixed equilibrium positions. No net long-distance transport is observed. This result confirms that the asymmetric periodic potential is an indispensable prerequisite for generating spontaneous directed transport in the particle chain; without the ratchet potential, directional motion cannot be sustained in the absence of external driving.

Subplot (c) adopts the parameters $\alpha =0.9,k=20,U_{0}=0.5,\Delta =1,a=0.45,D=0$. In contrast to subplot (a), all particles exhibit continuous negative displacement, which verifies the occurrence of reverse directed flow. The reverse transport arises from the matching of larger equilibrium spring length *a* and stronger coupling coefficient *k*. Under such parameter combinations, collective particle‑chain dynamics give rise to this observed reverse transport behavior.

Overall, the particle chain exhibits different dynamical behaviors under different parameter combinations: stable forward directed transport, pinned state without net directed transport, and reverse directed transport. The joint effect of coupling strength, equilibrium spring length and ratchet potential determines the occurrence and direction of the directed transport.

#### Influence of system parameters on average velocity of particle chain.

Fig 6 illustrates the dependence of the particle chain’s average velocity *v* on the system order α under different coupling coefficients. It can be observed that for suitable coupling strengths (e.g., *k* = 8,9,10), the average velocity generally rises as the system order increases. This indicates that the power-law memory originating from the fractional medium hinders the forward motion of the particle chain to some extent. Excessively strong memory confines the particle chain within the potential wells. Furthermore, weaker coupling strengthens the constraint exerted by the medium’s memory effect on the average velocity. When *k* = 5, the system remains stationary for all values of the system order. Therefore, asymmetric coupling acts as one of the key factors facilitating the emergence of directed transport. In addition, for *k* = 9 and α<0.6, the average velocity turns negative, meaning reverse flow arises in the particle chain when parameters including coupling strength and the medium’s memory effect meet certain matching conditions.

**Fig 6:**
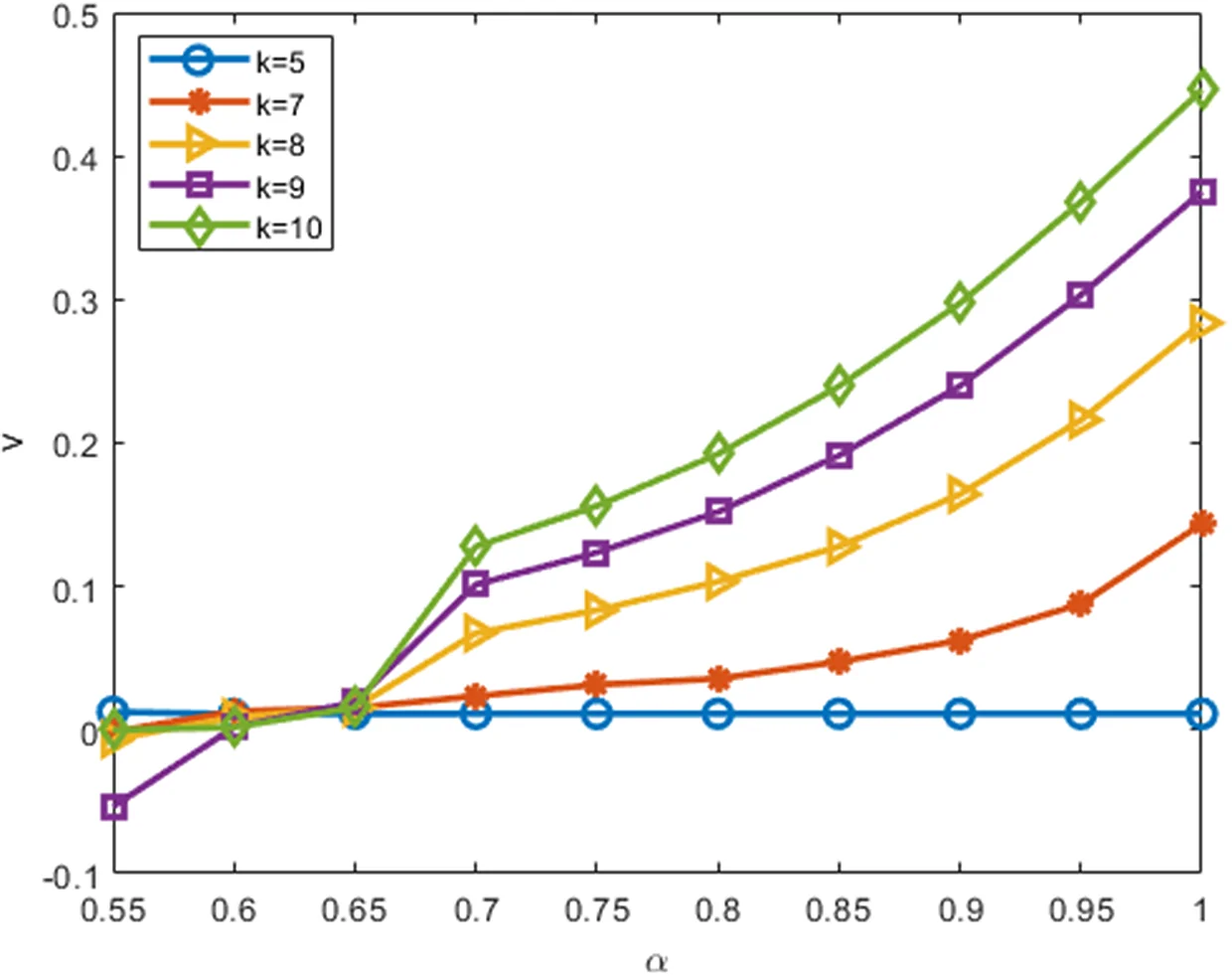
Average velocity of particle chains vs. system order under different coupling coefficients. Other parameters are set as follows: $N=10,U_{0}=0.5,\Delta =1,a=0.2,D=0$.

Fig 7 illustrates the dependence of the directed flow average velocity *v* on the particle chain’s coupling coefficient *k* for various equilibrium spring lengths and asymmetry parameters.

**Fig 7:**
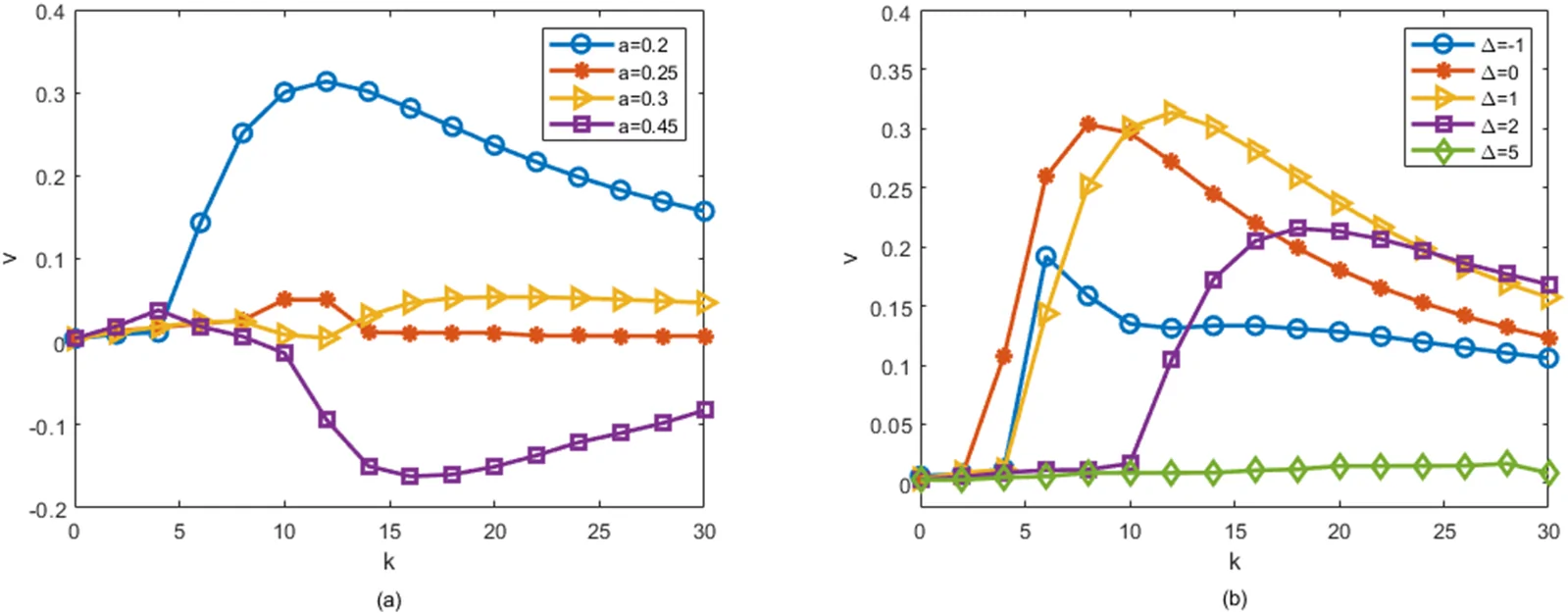
Average velocity of particle chains vs. coupling coefficient under different equilibrium spring length in (a) with Δ=1 and different asymmetry parameters in (b) with *a* = 0.2. Other parameters are set as follows: $\alpha =0.9,U_{0}=0.5,N=10,D=0$.

As can be seen from Fig 7(a), when the coupling coefficient *k* exceeds a certain critical threshold, the particle chain can cross the barriers of the ratchet potential and form stable directed flow. The direction of directed flow is closely related to the equilibrium spring length *a*: the flow is forward for *a* = 0.2, while reverse flow occurs for *a* = 0.45. The average velocity of the particle chain depends on both the coupling coefficient *k* and the equilibrium spring length *a*. For small values of *a*, the particle chain exhibits forward directed transport, and there exists an optimal coupling coefficient maximizing the forward average velocity, which manifests a generalized resonance phenomenon. The forward transport is gradually suppressed as *a* increases. When the equilibrium spring length rises to *a* = 0.45, the average velocity changes from positive to negative once the coupling coefficient exceeds a critical value, and reverse flow emerges in the system.

Physically, the parameter *a* determines the spatial arrangement of particles within the ratchet potential. A small *a* enables particle configurations compatible with the asymmetry of the potential, facilitating forward barrier crossing. When *a* is relatively large, the intrinsic interparticle distance mismatches the geometric characteristics of the ratchet potential. As the coupling strength increases, interparticle interactions are significantly enhanced, driving the particle ensemble to preferentially cross the potential barriers in the reverse direction.

In summary, with other parameters fixed, the occurrence of reverse flow is mainly determined by the equilibrium spring length and the coupling strength. Suitable combinations of these two parameters can reverse the intrinsic transport direction induced by the asymmetric periodic potential and produce reverse directed transport. Accordingly, one can regulate the magnitude and direction of the particle‑chain transport velocity by tuning the coupling strength and equilibrium spring length.

It can be seen from Fig 7(b) that the asymmetry parameter exerts a notable influence on the directed flow velocity. For the symmetric periodic potential (Δ=0), forward directed flow still emerges. In this case, the symmetry breaking generating directed flow originates from the asymmetric interparticle coupling, and the velocity varies non-monotonically with the coupling coefficient. When the periodic potential becomes asymmetric (Δ≠0), the tilt direction and magnitude of the potential barrier strongly affect the peak velocity *v*(*k*)_max_. Furthermore, the asymmetry degree governs the threshold coupling for triggering directed transport of the particle chain: a larger asymmetry magnitude |Δ| requires stronger interparticle coupling to sustain directed flow. If the asymmetry parameter is excessively large (Δ=5), the particle chain cannot surmount the potential barrier.

For a fixed equilibrium spring length and fixed asymmetry parameter, we separately investigate how the coupling coefficient affects the average velocity of the particle chain. It is found that the average velocity exhibits a generalized resonance phenomenon with respect to the coupling coefficient; namely, an optimal coupling strength exists to maximize the directed flow velocity. In other words, neither excessively weak nor excessively strong coupling facilitates particle transport.

Specifically, when the coupling coefficient *k* is too small, the interparticle interaction is insufficient to drive particles to cross potential barriers and establish directed flow. In the limiting case *k* = 0, all particles move independently, and the system reduces to a set of isolated single-particle systems. When *k* lies within an intermediate range, the average velocity of the particle chain first rises and then falls as the coupling coefficient increases. By contrast, when *k* becomes sufficiently large, interparticle interactions turn into mutual confinement, and the motion of each particle is heavily restricted by its neighbors. In the limit k→∞, the dynamical behavior of the system is equivalent to the motion of a single particle subjected to the effective potential $V_{\text{eff}}(x)=\frac{1}{N}\sum_{i=1}^{N}V(x+ia)$ [26,42].

Fig 8 displays the three-dimensional surface plot of the particle chain’s average velocity *v* as a function of the system order α and the asymmetry parameter Δ, with all other parameters fixed. For several selected values of the system order α, we extract two-dimensional curves of the average velocity versus the asymmetry parameter, as illustrated in Fig 9.

**Fig 8:**
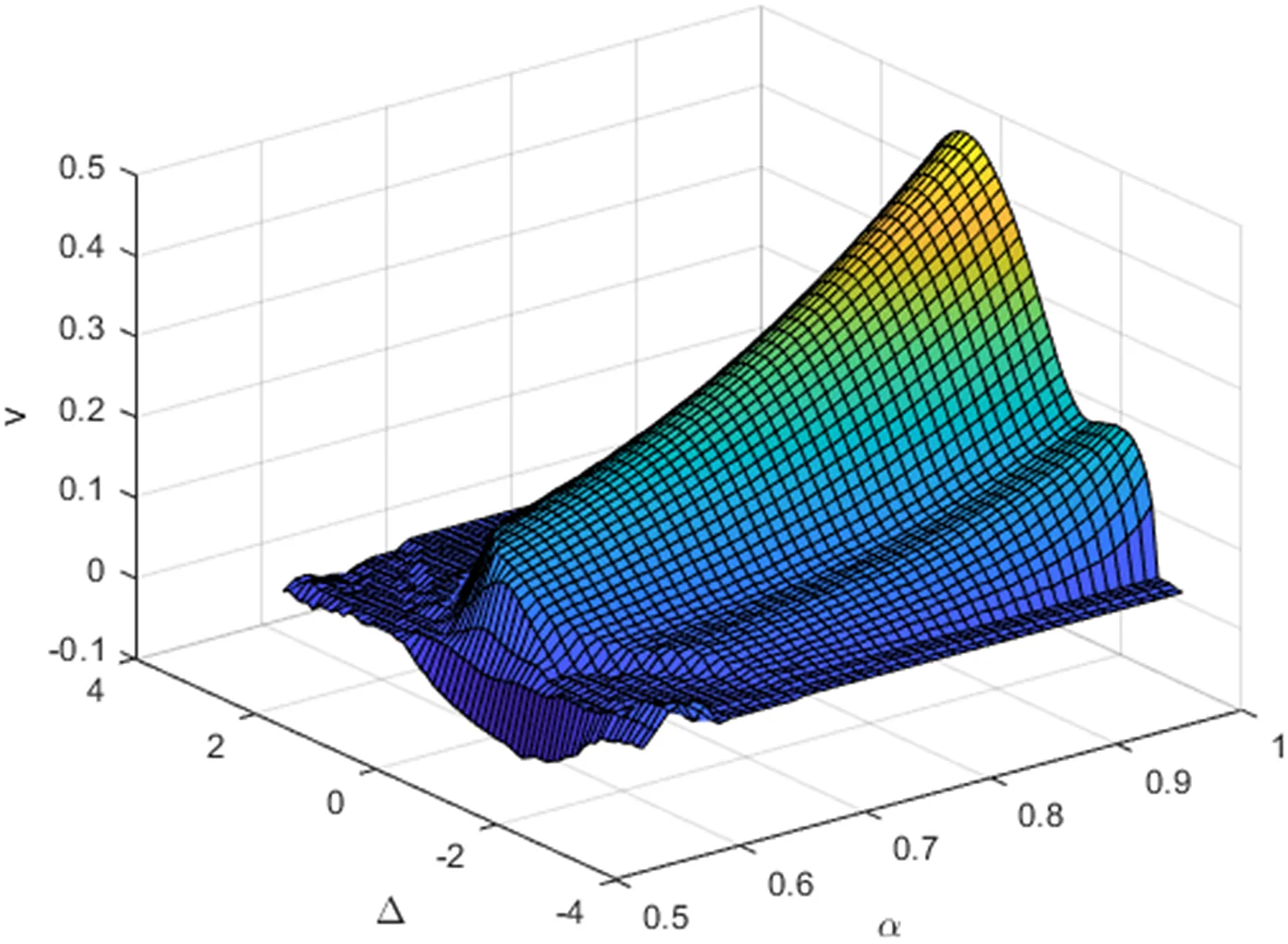
Average velocity of particle chains vs. system order and asymmetry parameter. Other parameters are set as follows: *N* = 10, *k* = 10, *U*_0_ = 0.5, *a* = 0.2, *D* = 0.

**Fig 9:**
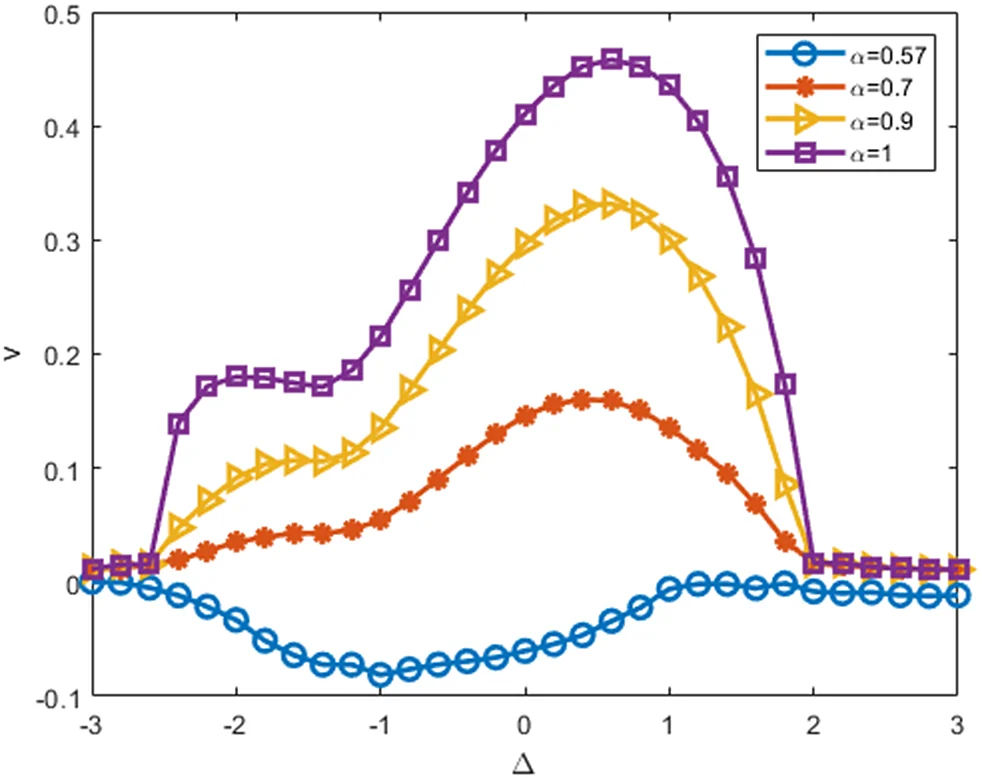
Average velocity of particle chains vs. asymmetry parameter under different system orders. Other parameters are set as follows: *N* = 10, *k* = 10, *U*_0_ = 0.5, *a* = 0.2, *D* = 0.

Fig 9 illustrates the variation of the average velocity of the particle chain with the asymmetry parameter Δ of the periodic potential under different fractional orders. The integer-order system with α=1 exhibits remarkable forward directed transport. As the fractional order α decreases, the memory effect of the medium is strengthened, and the forward average velocity declines continuously. When the memory effect becomes sufficiently strong (α=0.57), the average velocity turns negative for Δ<0, and reverse flow emerges in the system.

Physically, the parameter Δ governs the spatial asymmetric configuration of the ratchet potential, which creates the inherent difference in barrier hopping difficulty on either side. The fractional order α characterizes the medium memory property; smaller α implies that the particles’ historical motion imposes a stronger persistent influence on the current dynamics. For the integer-order system, potential asymmetry dominates particle transport and facilitates forward flow. When fractional memory effects are incorporated, cumulative historical dynamics hinder particles from moving along the transport direction favored by the ratchet potential and suppress forward directed transport. If the memory effect becomes sufficiently strong and the asymmetry parameter falls within an appropriate range, such temporal memory can offset the intrinsic transport bias originating from the potential, driving particles to cross barriers preferentially in the opposite direction and generating reverse flow. Such a mechanism is different from conventional integer-order ratchet models, where reverse flow is mainly induced by symmetry breaking of interparticle coupling or noise effects. Accordingly, the memory effect acts as an additional driving factor for reverse flow in the fractional framework. It can be concluded that reverse flow originates from the joint interplay between the asymmetry of the periodic potential and the medium memory effect.

Fig 10 depicts the dependence of the particle chain’s average velocity v on the asymmetry parameter Δ for various coupling coefficients. As shown in the figure, in the absence of interparticle coupling (*k* = 0), the average velocity satisfies v≡0, meaning all particles remain stationary at their initial positions. When interparticle coupling exists (k≠0), we obtain *v* > 0; due to mutual interactions, particles collectively move in the same direction and surmount the periodic potential barriers. This result reveals that without external force or noise excitation, directed flow cannot be guaranteed even when the periodic potential breaks symmetry (Δ≠0). Therefore, asymmetric interparticle coupling serves as a necessary condition for the emergence of directed flow in this deterministic system.

**Fig 10:**
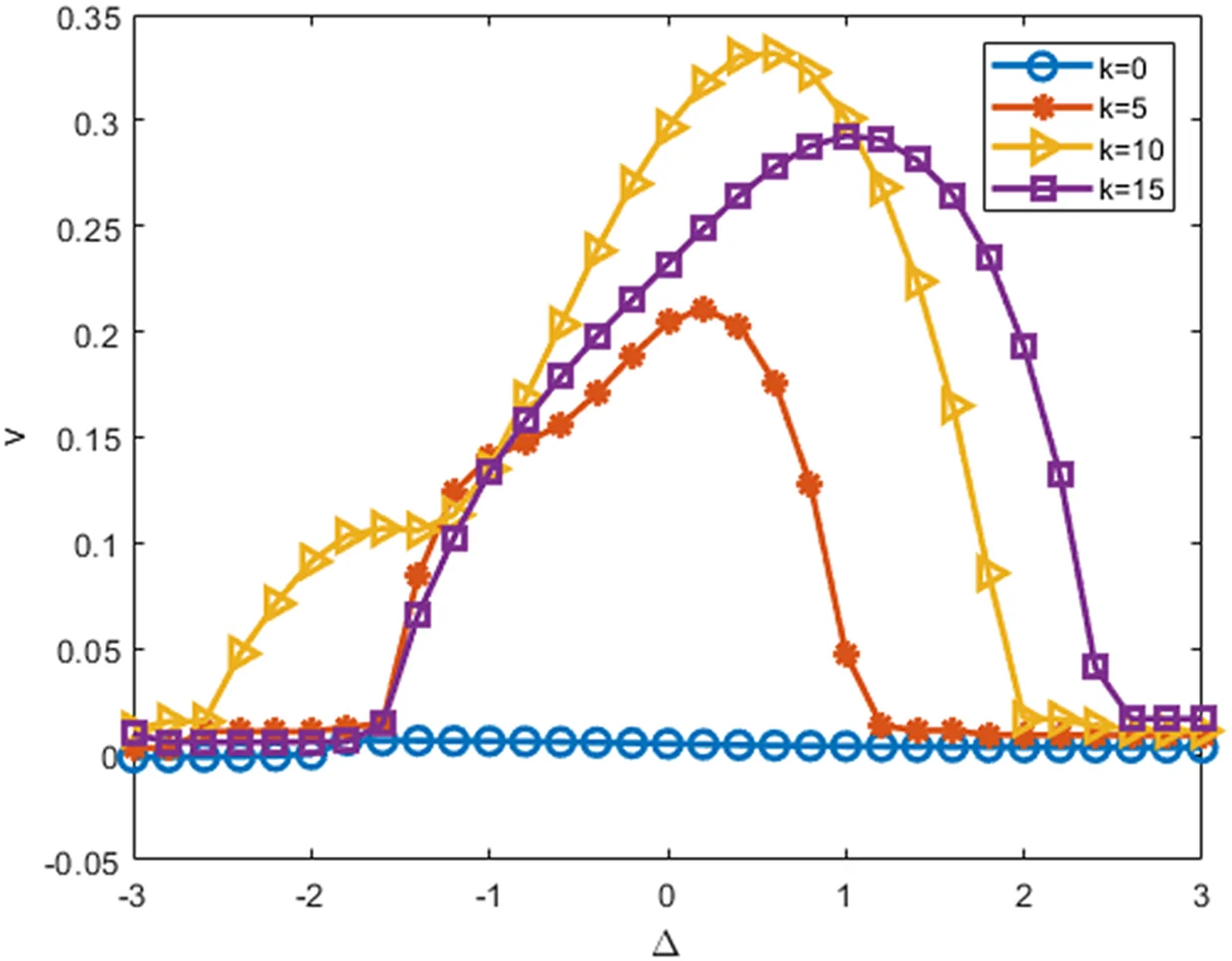
Average velocity of particle chains vs. asymmetry parameter under different coupling coefficients. Other parameters are set as follows: $\alpha =0.9,N=10,U_{0}=0.5,a=0.2,D=0$.

In addition, for a fixed coupling coefficient, when the asymmetry of the periodic potential lies within a certain range, the average velocity *v* of the particle chain varies non-monotonically with the asymmetry parameter Δ, indicating a generalized resonance phenomenon. Beyond this range, the particle chain remains in a pinned state. It follows that stronger asymmetry of the periodic potential does not always yield better transport performance; excessive asymmetry of the periodic potential suppresses the directed transport of the particle chain.

Fig 11 illustrates how the particle chain’s average velocity *v* varies with the barrier height *U*_0_ under different asymmetry parameters. It can be observed that, for a fixed asymmetry parameter, directed flow can be generated as long as the periodic potential is present. Moreover, the average velocity exhibits generalized resonance with respect to the barrier height. When the barrier height exceeds a critical threshold, pinning occurs and the particle chain ceases net motion. This critical threshold depends strongly on the asymmetry parameter. For instance, the critical value reads $U_{0}^{*}=0.5$ for Δ=2, whereas $U_{0}^{*}=1.5$ for Δ=0. The underlying mechanism governing this complicated parametric dependence remains to be further explored.

**Fig 11:**
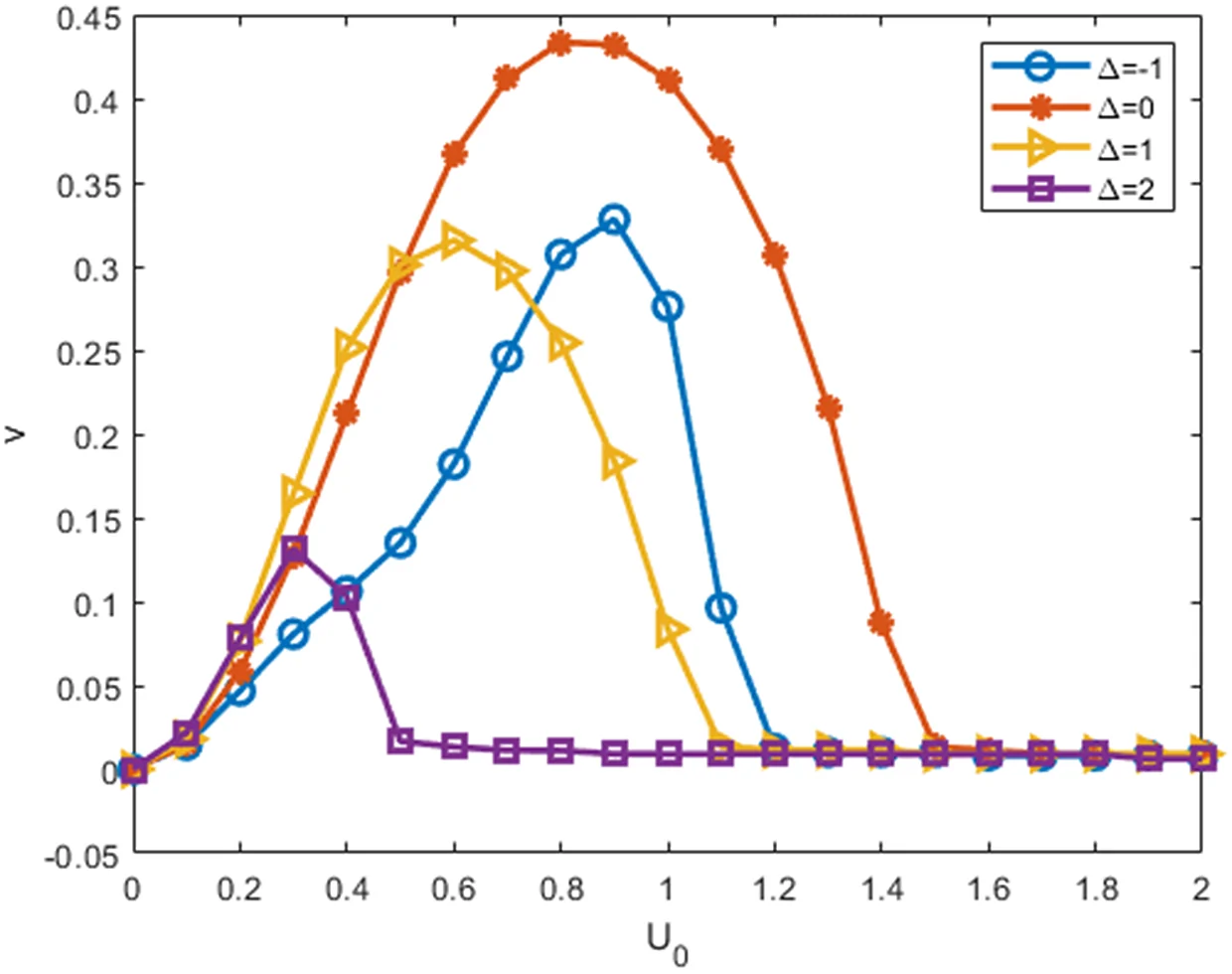
Average velocity of particle chains vs. barrier height under different asymmetry parameters. Other parameters are set as follows: α=0.9,N=10,k=10,a=0.2,D=0.

The system governed by model (Eq (9)) involves two spatial scales: the equilibrium spring length *a* and the spatial period *L* of the ratchet potential. The cooperation and competition between these two scales give rise to rich transport behaviors. Fig 12 illustrates the dependence of the particle chain’s average velocity v on the equilibrium spring length *a* under different barrier heights. It can be observed that all curves exhibit approximate antisymmetry about *a* = 0.5, namely v(a)≈−v(1−a). Deviations from exact antisymmetry originate from the spatial inversion asymmetry of the ratchet potential. For equilibrium spring length satisfying a∈(0,0.5], forward directed flow dominates the dynamics of the particle chain, whereas reverse directed flow prevails for a∈(0.5,1]. Nevertheless, at certain barrier heights (e.g., *U*_0_ = 0.2), reverse flow can be detected even in the region *a* < 0.5.

**Fig 12:**
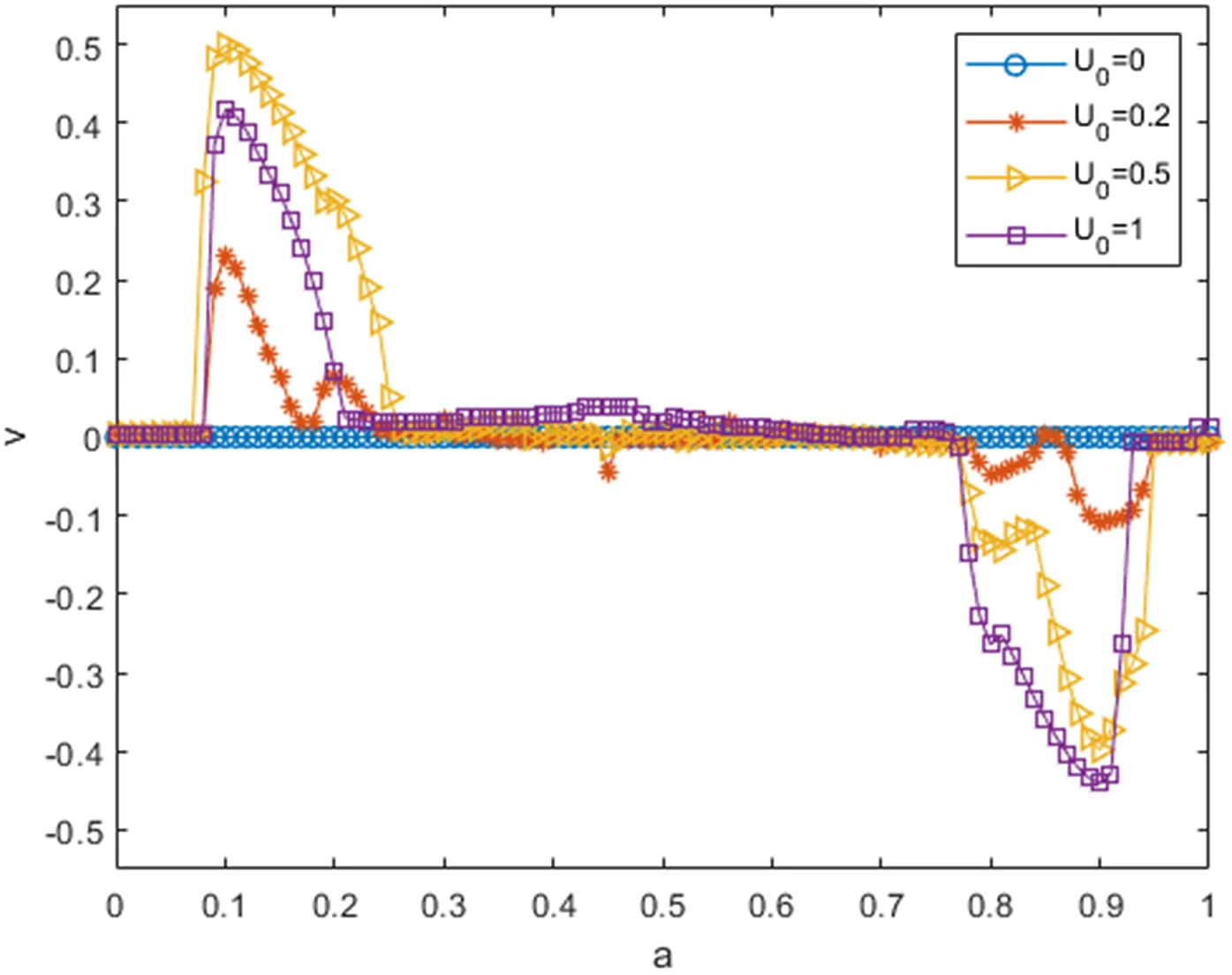
Average velocity of particle chains vs. equilibrium spring length under different barrier heights. Other parameters are set as follows: α=0.9,N=10,k=10,Δ=1,D=0.

For the equilibrium spring length satisfying a∈(0,0.5], the average velocity *v* of the particle chain varies non-monotonically with *a*, featuring a resonance peak. At appropriate barrier heights, the curve *v*(*a*) further exhibits double-peak resonance or even multi-peak resonance. Accordingly, similar to the cases of *v*(*k*) and *v*(*U*_0_), the equilibrium spring length *a* can serve as an optimization parameter to maximize the directed flow velocity *v*.

### Directed transport in the presence of noise (D≠0)

Fig 13 compares the displacements of the particle chain obtained with and without noise. It can be seen that the particle chain undergoes no directed motion in the noise-free case. By contrast, reverse directed flow emerges when noise of finite intensity is introduced into the system. This indicates that noise plays a constructive role in facilitating particle transport under these parameter conditions.

**Fig 13:**
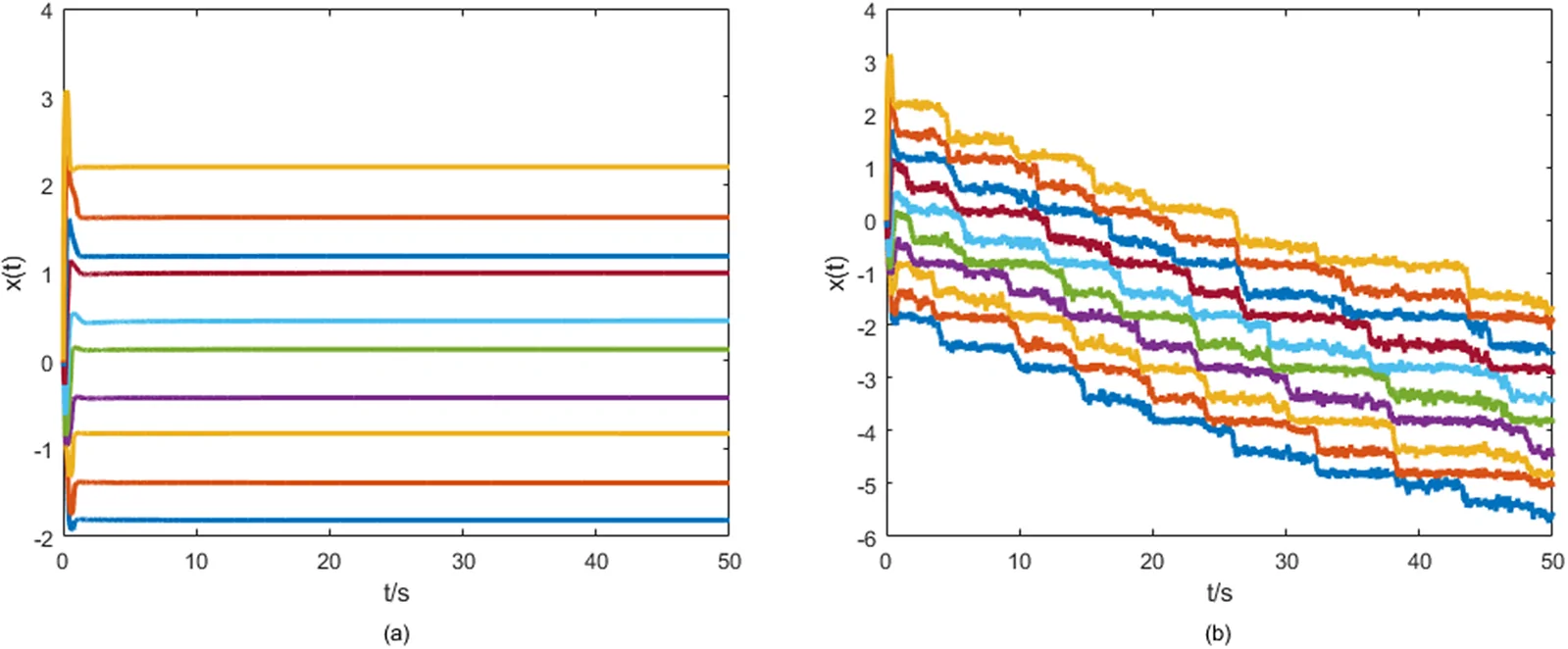
Displacement plot *x*(*t*) of particle chains under asymmetry parameters. (a): $\alpha =0.9,k=10,\Delta =1,a=0.45,U_{0}=0.55,D=0$ and (b): $\alpha =0.9,k=10,\Delta =1,a=0.45,U_{0}=0.55,D=2$.

Fig 14 illustrates the dependence of the particle chain’s average velocity *v* on the noise intensity *D*. The average velocity exhibits a non‑monotonic dependence on noise intensity, demonstrating the feature of stochastic resonance: moderate noise facilitates net directed transport, whereas excessively strong noise suppresses the transport performance. Meanwhile, both forward and reverse directed flows can be observed in the system. With other parameters kept fixed, the flow direction mainly depends on the equilibrium spring length, which is consistent with the previous discussion for the noise‑free case.

**Fig 14:**
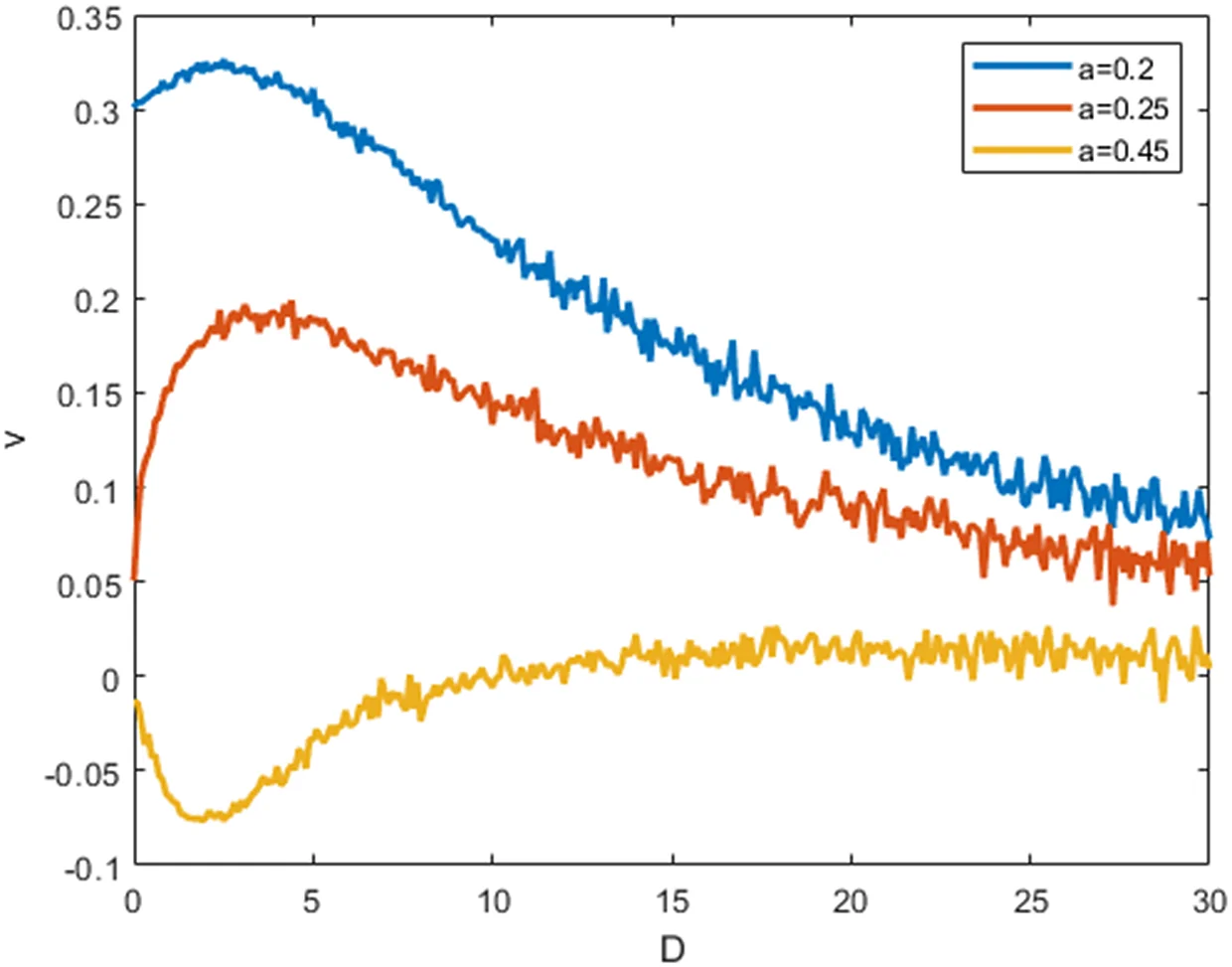
Average velocity of particle chains vs. the noise intensity under equilibrium spring length. Other parameters are set as follows: $\alpha =0.9,N=10,k=10,U_{0}=0.5,\Delta =1$.

## Conclusion

To summarize, this paper employs fractional calculus to describe the power-law memory effect of the system and establishes a mathematical model for a fractional-order over-damped Brownian motor subjected to an asymmetric periodic potential and asymmetric interparticle coupling. The fractional difference scheme is adopted to numerically simulate the proposed model, and the cooperative directed transport behaviors of this fractional nonlinear system are investigated. We analyze and discuss how the coupling strength, system order, barrier height, asymmetry parameter, equilibrium spring length, and noise intensity affect the directed flow velocity.

Our main conclusions are listed as follows:

1. Numerical results reveal that directed transport can be achieved in the fractional-order system with asymmetric coupling without external forcing (both deterministic and stochastic forces). Under fixed remaining parameters, the directed transport velocity of the particle chain rises as the fractional order increases.
2. With suitable parameter settings, either forward or reverse directed flow emerges in the particle chain. The directed flow velocity depends on the barrier height, coupling coefficient, equilibrium spring length and other control parameters and varies non-monotonically. Such dependence exhibits generalized single-peak, double-peak, and even multi-peak resonance.
3. For fixed parameters, the curve *v*(*a*) describing the average velocity versus the equilibrium spring length satisfies approximate antisymmetry about *a* = 0.5.
4. When noise is introduced into the system, stochastic resonance occurs, whereby noise enhances the directed transport velocity under suitable system parameters.

We note that this study is limited to theoretical and numerical analysis, and we leave the corresponding experimental verification as an important direction for our future research.

## Supporting information

S1 Dataset Raw simulation data underlying all figures in the manuscript. This compressed zip archive contains the raw data files used to generate each figure. All simulation parameters are fully described in the main text.
(RAR)
